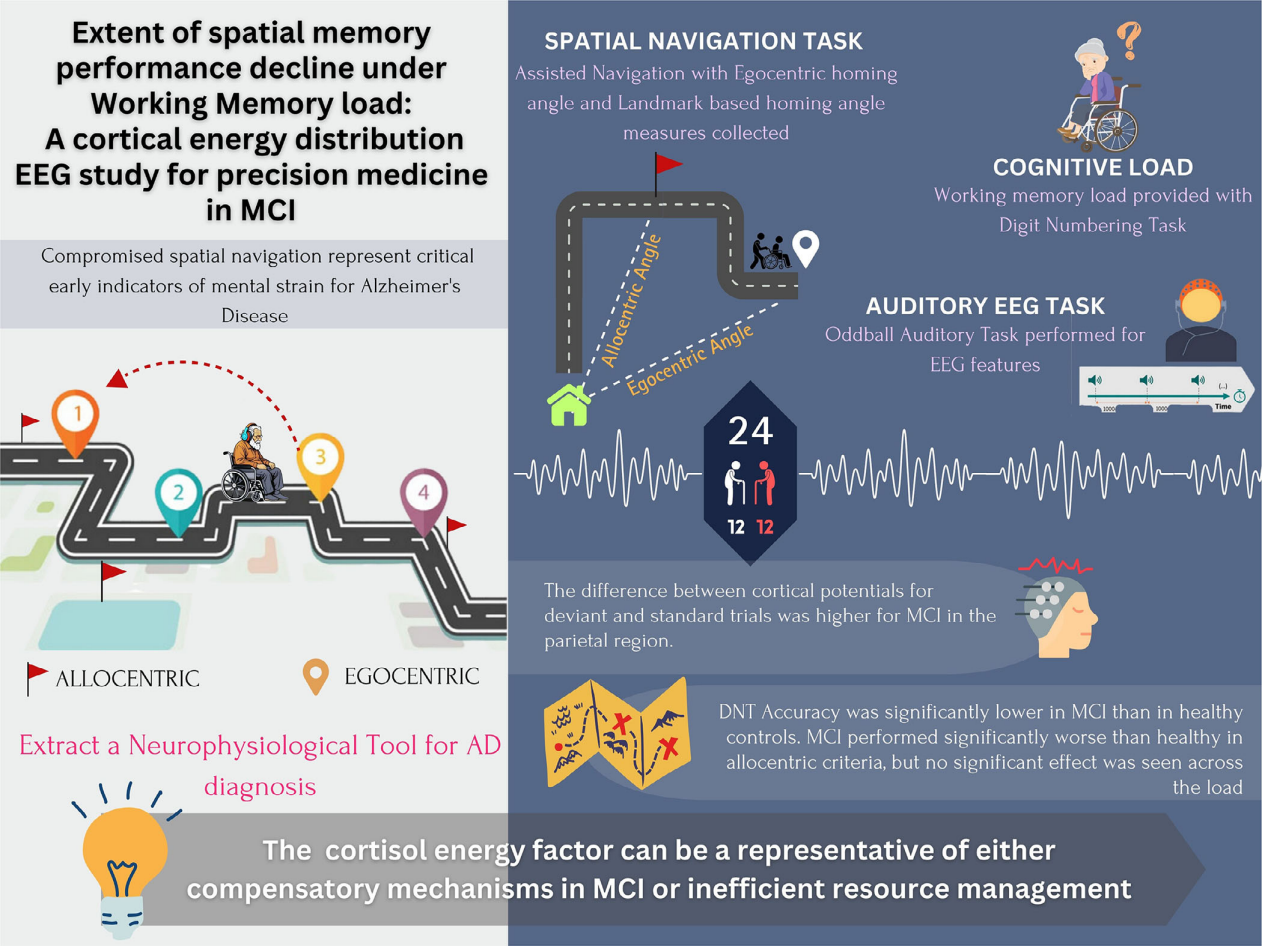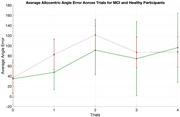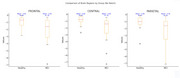# Disentangling the loading values of Ego‐ versus Allo‐centric navigation performance, Working memory, Error processing based cognition and cortical energy distribution for precision medicine in mild cognitively impaired patients

**DOI:** 10.1002/alz70856_104124

**Published:** 2025-12-24

**Authors:** Shreelekha BS, Adrija Chatterjee, Pragathi Balasubramani

**Affiliations:** ^1^ Translational Neuroscience and Technology Lab, Indian Institute of Technology Kanpur, Kanpur, Uttar Pradesh, India

## Abstract

**Background:**

Compromised object‐based spatial navigation and spatio‐cognitive map navigation are critical early indicators of Alzheimer's Disease. Distinct neural processes, such as the interaction between posterior parietal cortices and lateral entorhinal cortex, are significant for egocentric object‐based processing, while the retrosplenial cortex (RSC) and hippocampus interaction is important for allocentric map processing. Identifying the mechanisms underlying cognitive impairment can help engage dysfunctional circuits and improve rehabilitation through closed‐loop stimulation paradigms. In this study, we explore these circuits with a passive cognitive task that evaluates cortical energy distribution and increases working memory load, while participants navigate in real‐world settings. We hypothesize that ego‐ versus allocentric navigation performance decline under high memory load may reveal early damage in relevant circuits, which could serve as clinical markers.

**Method:**

**Participants navigated five standardized paths with varying stress loads (high and low) while performing a Digit Numbering Task (DNT). An auditory mismatch negativity paradigm was also used to assess cortical energy distribution. Behavioral data were collected with electroencephalography for *N* = 24 (age>55, 14 males, MCI=12) subjects, screened as control or MCI using the Mini‐Mental State Exam**.

**Results:**

A significant difference was observed in DNT accuracy between groups (control: 68.9±38.8, MCI=52.3±42.1, *p* =  0.04*). No significant differences were found between the two groups for egocentric or allocentric performance across working memory loads. However, allocentric performance was significantly worse in MCI (67.14±54.47, 88.96±53.23, *p* =  0.0013**). The energy factor, measured as the difference in cortical potentials for deviant and standard trials, was higher for MCI in the parietal region (mu for control= ‐1.016, MCI= ‐2.34 microV, *p* =  0.0036).

**Conclusion:**

The poorer allocentric performance in MCI suggests RSC involvement, offering a potential biomarker for closed‐loop stimulation. Additionally, the increased energy factor observed in the parietal area may indicate compensatory mechanisms in MCI or inefficient resource management. Future work will validate these findings using high‐resolution source localization methodologies.